# Self-Concept in Primary School Student with Dyslexia: The Relationship to Parental Rearing Styles

**DOI:** 10.3390/ijerph18189718

**Published:** 2021-09-15

**Authors:** Anyan Huang, Mingfan Sun, Xuanzhi Zhang, Yuhang Lin, Xuecong Lin, Kusheng Wu, Yanhong Huang

**Affiliations:** 1 Mental Health Center, Shantou University Medical College, North Taishan Road, Shantou 515065, China; 19ayhuang@stu.edu.cn (A.H.); zhangxuanzhi2018@163.com (X.Z.); 18yxlin@stu.edu.cn (Y.L.); alinxuecong@126.com (X.L.); 2 Department of Preventive Medicine, Shantou University Medical College, Shantou 515041, China; 18mfsun@stu.edu.cn

**Keywords:** dyslexia, self-concept, parental rearing style, correlation

## Abstract

Dyslexic children may be more likely to form a negative self-concept, especially with poor educational experiences and negative parenting. The purpose of the present study was to investigate the self-concept of Chinese dyslexic children in primary school, and explore the influence factors of self-concept as well as its relationship with parenting style. A total of 50 children with dyslexia and 50 non-dyslexics matched for age, grade and gender participated in the study. We used the Piers-Harris children’s self-concept scale (PHCSS) and the Chinese version of Egna Minnen Beträffande Uppfostran for Children (EMBU-C) to evaluate the self-concept and parenting styles of the study population. Our results indicated that the academic competence, popularity and general self-concept in the dyslexic group were significantly lower than those in the control group (*p* < 0.05). Based on the multivariate linear regression, we also found that residence (*β* = −0.32, *p* < 0.05) and physical activity (*β* = 0.36, *p* < 0.01) may influence factors self-concept in dyslexic children. In addition, a moderate and positive correlation was found between the self-concept of physical appearance and maternal emotional warmth (*r* = 0.36, *p* < 0.05) by using the Spearman correlation analysis. Our outcomes suggested that children with dyslexia have a poorer self-concept than typical developing children. The self-concept of dyslexic children should be improved in order to achieve better physical and mental development.

## 1. Introduction

Developmental Dyslexia (DD), characterized by inaccurate, slow or laborious reading, as well as poor spelling, is a neurodevelopmental disorder caused by a variety of genetic, neurological and cognitive factors [1]. Despite adequate educational experience and the lack of significant sensory or neurological deficits, children with DD still do not acquire the same effective reading and spelling skills as normally developing children [2]. The latest epidemiology study reported that dyslexia is highly prevalent, affecting 20% of the population [3], while in China, the prevalence of dyslexia ranges from 3% to 10% [4,5,6]. Our previous epidemiological investigation also showed that the prevalence of dyslexia among children in Shantou was 5.4% [7]. Unfortunately, the majority of teachers or parents from China did not know about dyslexia, even though it has been studied domestically for more than a decade. Although the prevalence of dyslexia in Chinese does not appear to be as high as in other languages, the influence of Chinese dyslexia still needs to be taken into account. A cost-benefit analysis from the UK suggested that people who suffered from dyslexia could lose more than £80,000 of their potential life-time earnings [8]. However, the most immediate impact of dyslexia on children may be the phenomenon of school failure. These academic setbacks will destroy the self-confidence of children and directly impede the formation of a positive self-concept. Moreover, a qualitative analysis of interviews with children with poor reading found that the negative feedback from peers, teachers and parents may also lead them to develop a negative self-concept [9], which can affect children’s mental health, leading to a variety of emotional problems.

Self-concept is the totality of one’s own cognitive image, namely one’s experience of one’s own existence [10]. It is a cognitive mechanism composed of attitudes, emotions, beliefs and values, which involves a gradual deepening of one’s understanding of oneself through experience, reflection and feedback from others. Self-concept can be divided into general/global self-concept (sometimes expressed as self-esteem) and self-concept in different fields [11]. For instance, the domains of self-concept include behavior problem self-concept, academic competence self-concept, physical appearance self-concept, and so on. The formation of self-concept is an important aspect of children’s socialization. Guiding children to form a positive self-concept from the beginning should be a pre-determined educational orientation. Studies have shown that a positive self-concept in children was related to improved academic performance, effective use of coping skills, and safe and healthy social relationships [12]. Similarly, a strong and stable self-concept is also important to children’s cognitive, emotional and psychosocial development [13]. On the contrary, a negative self-concept can not only trigger negative self-expectations of children, but also cause many emotional problems, such as depression and anxiety [14]. These negative emotions may in turn affect the self-concept of children, forming a vicious circle. Moreover, peer relationship and school environment are also important factors affecting self-concept in children [15,16]. Especially for children with dyslexia, they are often misunderstood by their peers and teachers due to their academic failure. Poor grades and negative feedbacks from others could reinforce their negative self-concept. Several studies have investigated and analyzed the self-concept of people with poor reading skills. McArthur et al., found that, poor readers were at higher risk for low academic and general self-concept [17]. Based on the above research, we hypothesized that dyslexic children have lower self-concept than normal children in China, especially academic self-concept. However, research on self-concept in Chinese dyslexic children was scarce. Children with Dyslexia in Chinese may receive different feedback from the external environment than children in other countries because of the differences in educational patterns between different cultures, which may affect their self-concept. Therefore, it is necessary to investigate the self-concept of Chinese dyslexic children in order to evaluate their mental health level.

In addition to feedback from teachers and peers that can influence the self-concept of children, parental rearing styles are equally important. Research has shown that among the various family factors that may affect children’s development, parental rearing style was considered to be highly significant [18]. Parental rearing style can be summarized as the behaviors, attitudes and values adopted by parents when they interact with their children, which affect the physical, emotional, social and intellectual development of the children [19]. Some studies have shown that general self-concept was influenced by different rearing styles of parents [20,21]. In particular, children with dyslexia were usually faced with parents’ incomprehension. The persistent academic failure of dyslexic children may cause parents to adopt a negative rearing style, which further affects the development of positive self-concept. Nevertheless, according to our knowledge, no research has been found on the relationship between self-concept and parental rearing styles in Chinese dyslexic children. Hence, it is of great significance to explore the relationship between the self-concept of children with dyslexia in China and their parental rearing style.

Children with poor self-concept are also part of a hidden crisis, as it is a predictor of poorer mental health, antisocial behavior, anxiety, depression and suicidal tendency [22,23]. Children who have a low self-concept are also more likely to commit crimes as young adults [24]. By reason of the foregoing, we investigated the self-concept of Chinese dyslexic children, the influencing factors and the relationship between self-concept and parental rearing style. The purpose of the present study was to provide information on psychological intervention strategies for dyslexic children in China, in order to reduce the incidence of high-risk behaviors and poor mental health caused by negative self-concepts.

## 2. Materials and Methods

### 2.1. Study Participants

The case group of the current study was comprised of children diagnosed with dyslexia from May to October 2020 in the dyslexia specialist clinic of the Mental Health Center, Shantou University Medical College. A total of 56 children were diagnosed with dyslexia during that period. The diagnostic criteria of the case group were consistent with our previous studies [7]. Briefly, we followed the including criteria as: (1) IQ above 80 by the Raven’s Standard Progressive Matrices; (2) at least 1 standard deviation below the average level of their actual grade on the Chinese Vocabulary Test and Assessment Scale, which is the widely used reading level test for screening Mandarin-speaking Chinese children for dyslexia (Cronbach’s α = 0.75) [25]; (3) according to the reports from their head-teachers, Chinese language test had been below 10% among all children in the same grade for 6 months; (4) there were at least 2 standard deviations above the mean score in the Dyslexia Checklist for Chinese Children (DCCC), which has a good reliability for Chinese dyslexia identification (Cronbach’s α = 0.97) [26]; (5) no suspected brain damage, uncorrected sensory impairment, other mental or neurological disorders or any comorbidities (e.g., attention deficit hyperreactivity disorder); (6) child psychiatrists make the final diagnosis according to the diagnostic criteria of the fifth edition of Diagnostic and Statistical Manual of Mental Disorders (DSM-5), combined with the Chinses Reading Ability Test (CRAT), which is used for auxiliary diagnosis (Cronbach’s α = 0.75) [27,28]. After a detailed introduction to our study, we invited these children and their parents to fill out questionnaires. A total of 50 children with dyslexia and their parents were eventually enrolled in this study after giving written informed consent.

The children in the control group were recruited from a randomly selected primary school in Shantou. The case and control groups were matched for age, grade, and gender by a ratio of 1:1. We followed the including criteria as: (1) normal children without dyslexia; (2) no history of neurological diseases or psychiatric disorders; (3) normal or corrected-to-normal vision; (4) normal IQ; (5) agreed to participate in this research and signed the written informed consent.

### 2.2. Instruments

#### 2.2.1. Piers-Harris Children’s Self-Concept Scale (PHCSS)

The PHCSS is a self-rating scale for children developed by Piers E. and Harris D. in 1969 and revised in 1974 [29]. It is mainly used to evaluate self-concept (also known as self-awareness) of children and adolescents aged 8 to 16 years. Su et al., revised the PHCSS and developed the urban norm in China [30]. The PHCSS consists of 80 items which are scored true or false, and can divided into 6 subscales: behavior problems, academic competence, physical appearance, anxiety, popularity and happiness. The total score of PHCSS is 80, which reflected the general level of self-concept of the participants. The higher the score on each subscale, the better the self-concept in each dimension. It has good reliability and validity and the Cronbach’s α of the scale was 0.86. According to the provisions of the original scale, the total score between the 30th and 70th percentiles is the normal range; a score lower than the 30th percentile (equivalent to a score of 46) indicates a low level of self-concept, suggesting that the child may have some emotional or behavioral problems or social maladjustment, and have a tendency to lack self-confidence, and experience self-deprecation or self-abandonment. Scores higher than the 70th percentile (equivalent to a score of 58) indicates a higher level of self-concept, suggesting that the child may be demanding too much of himself and have an insufficient tolerance to setbacks.

#### 2.2.2. Egna Minnen Beträffande Uppfostran for Children (EMBU-C)

The EMBU-C is an effective scale which is used to evaluate the current parental rearing style of children. In our study, the Chinese version of EMBU-C which was translated and revised by Liming Tie et al., was used [31]. The Cronbach’s α of the scale was 0.82 and the retest reliability was between 0.72 and 0.90. The EMBU-C includes 4 dimensions, namely emotional warmth, rejection, overprotecting and anxious rearing. Each dimension consists of 10 items and is rated on a four-point Likert scale from never (0 point) to always (4 point). The participants had to answer each item twice to evaluate their current paternal and maternal rearing styles, respectively. The higher the score on one of the dimensions, the greater the propensity for the parental rearing styles represented by that dimension.

#### 2.2.3. The Chinese Reading Ability Test (CRAT)

The CRAT, which has a good reliability and validity (Cronbach’s α = 0.75), was established for auxiliary diagnosis of Chinese dyslexia. It consists of five subscales, respectively the phonological awareness subscale (PA), morphological awareness subscale (MA), rapid automatized naming subscale (RAN), orthographic awareness subscale (OA), and reading ability subscale (RA) [28]. The PA consists of three subtests measuring onset, rhyme and tone, respectively. The MA requires children to match characters in the first column with characters in the second column to form a meaningful compound word, and record the completion time and score. In the RAN, children are asked to name numbers from left to right, top to bottom, twice, as quickly and accurately as possible. The OA reflects the children’s knowledge of Chinese character structure and the sensitivity of students in distinguishing between characters and noncharacters. The RA included reading time, as well as oral and written questions. The test takes about 45 min. The scores of the five subscales of CRAT were calculated to identify whether there were differences in reading ability between dyslexic children and normally developing children.

### 2.3. Study Process

Data collection for the case group was performed in the dyslexia specialist clinic of the Mental Health Center, Shantou University Medical College. The professional investigators guided the children and parents to fill out the questionnaire after explaining the purpose of the study in detail and obtaining their written informed consent. In the course of the survey, if the participants encountered any questions that they did not understand, the investigators would make clear explanations. At the end of the survey, the investigator collected the questionnaires and checked the completion status, found out missing items, and reminded the participants to add these.

The data for the control group was collected in a public primary school in Shantou, with the assistance of the teachers. An envelope containing the writen informed consent and the questionnaires filled in by parents was given to the selected students by their teachers. They were asked to bring the envelopes to their parents on the same day and take them back on the next day. Based on the informed consent, the investigators conducted a survey in a classroom on the students whose parents agreed to take part in the research. Any omissions in the questionnaires filled out by parents was supplemented by our investigators through telephone interviews. The quality control process of the questionnaire filling for students in the control group was in accordance with the process for the case group.

### 2.4. Statistical Analysis

Data management software EpiData 3.1 (EpiData Version 3.1, EpiData Association, Odense, Denmark) was used to establish the database independently by two professionals. The consistency test in this software was used to check the consistency of the data. Quantitative variables were expressed as means ± standard deviations ($\bar{x}\pm s$), while qualitative variables were expressed by frequency and percentage. Before data analysis, Shapiro-Wilk normality test was used to test for normal distribution of the data. The paired *t* test was used to analyze the differences in continuous variables between the case group and the control group, and the McNemar test or Bowker test was used to analyze the differences in composition ratio. The factors that may have influenced self-concept of children were then evaluated using multiple linear regression models. The correlation between self-concept and parental rearing style was calculated using Spearman correlation analysis. All statistical analysis was performed using R 4.1.0 (R foundation, Vienna, Austria) and GraphPad Prism software (GraphPad Prism 9, Inc., San Diego, CA, USA). The significant level was set to 0.05.

## 3. Results

### 3.1. Demographic Characteristics

A total of 50 children with dyslexia and 50 typical developing children who were 1:1 matched with the case group in gender, age and grade were included in the study. The average age of the participants in both groups was 9.18 ± 1.37 years old. The dyslexic group included 37 boys and 13 girls. The distribution among grades and other characteristics of two groups is shown in Table 1. We found statistical differences between two groups in maternal education level (*χ*^2^ = 8.695, *p* = 0.034) and monthly family income (*χ*^2^ = 14.364, *p* = 0.002). Compared with the control group, the proportion of children in the dyslexic group who were scolded by their parents and had difficulty in doing homework was much higher (all *p* < 0.001).

### 3.2. The Results of the CRAT between the Dyslexic Group and the Control Group

As shown in Table 2, the CRAT showed significant statistical difference between the two groups (all *p* < 0.05). The children in the control group scored higher than those in the dyslexic group on all subscales. By contrast, children with dyslexia took longer to complete the test and scored lower.

### 3.3. Comparison of Self-Concept

**Figure 1:**
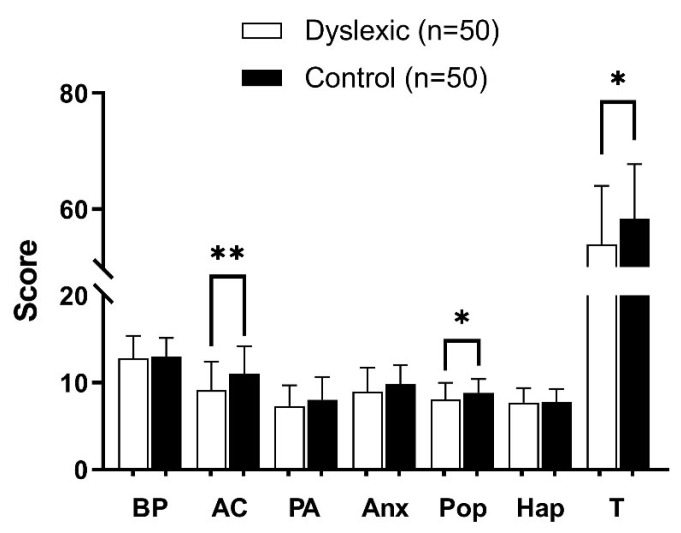
Difference in self-concept score between dyslexic group and control group. Abbreviations: BP, behavior problems; AC, academic competence; PA, physical appearance; Anx, anxious; Pop, popularity; Hap, happiness; T, total self-concept. Note: * *p* < 0.05; ** *p* < 0.01.

The self-concept scores of children in the dyslexic group and the control group are shown in Figure 1. The results show that there were statistical differences in the total score of self-concept between the two groups (*p* < 0.05). The scores for behavior problems, academic competence, physical appearance, anxiety, popularity and happiness in the dyslexic group were lower than those in the control group. Among them, the differences between academic competence (*p* < 0.01) and popularity (*p* < 0.05) in the two groups were statistically significant, while no statistical difference was found in behavior problems, physical appearance, anxiety and happiness (all *p* > 0.05).

According to the classification method mentioned above, the total score for self-concept was divided into three categories: low level of self-concept (≤ 45), normal level of self-concept (46 to 58) and high level of self-concept (≥59). There were significant differences in the composition ratio of self-concept level between the dyslexic group and the control group (Table 3). We further calculated the difference in the composition ratio of self-concept at different levels between two groups. The results showed that the composition ratio of children with low self-concept in the dyslexic group was higher than that in the control group, and the difference was statistically significant (*p* < 0.05).

### 3.4. Influence Factors of Self-Concept in Dyslexic Children

Multivariate linear regression analysis was conducted with the total score for self-concept of children with dyslexia as the dependent variable, and variables that may affect children’s self-concept as the independent variables. Stepwise method was selected for multivariate linear regression. Collinearity diagnosis showed no multicollinearity among included variables. As shown in Table 4, participation in physical activity and place of residence were influential factors for self-concept of children with dyslexia, which accounted for 27% of the total variation. The effects of other variables did not show statistical significance (all *p* > 0.05).

### 3.5. Comparison of Parental Rearing Style

**Figure 2:**
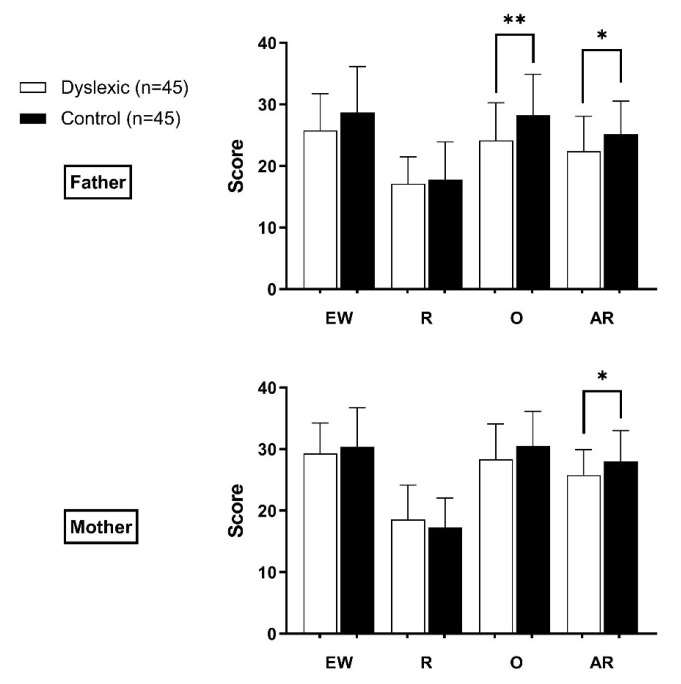
Difference of parental rearing style between dyslexic group and control group. Abbreviations: EW, emotional warmth; R, rejection; O, overprotecting; AR, anxious rearing. Note: * *p* < 0.05; ** *p* < 0.01; This part of the data analysis excluded children from a single parent family.

The scores of different types of parental rearing styles for children with dyslexia and children in the control group are shown in Figure 2. In the results of paternal rearing style, the scores for overprotecting and anxious rearing in the dyslexic group were lower than those in the control group (*p* < 0.05). Among the maternal rearing style, only anxious rearing had a statistically significant difference between the two groups (*p* < 0.05), with the dyslexic group scoring lower than the control group. No statistical differences were found in the scores of other parental rearing styles between two groups (*p* > 0.05).

### 3.6. Self-Concept and Parental Rearing Style

**Figure 3:**
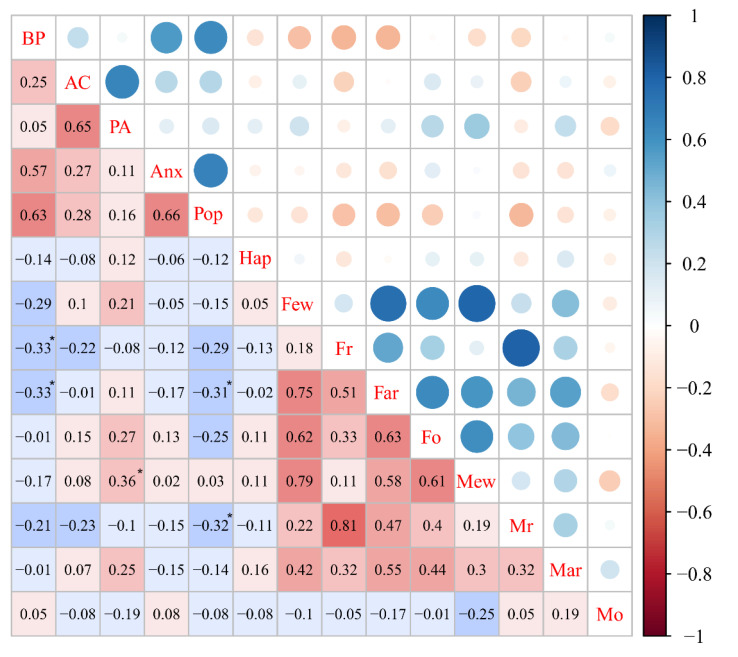
The relationship between self-concept and parental rearing style in the dyslexic group. Abbreviations: BP, behavior problems; AC, academic competence; PA, physical appearance; Anx, anxious; Pop, popularity; Hap, happiness; Few, father’s emotional warmth; Fr, father’s rejection; Far, father’s anxious rearing; Fo, father’s overprotection; Mew, mother’s emotional warmth; Mr, mother’s rejection; Mar, mother’s anxious rearing; Mo, mother’s overprotection. Note: * *p* < 0.05; Red for positive correlation, blue for negative correlation; the stronger the correlation, the darker the color.

The Spearman correlation analysis indicated that self-concept in children with dyslexia were related to their parental rearing styles. As shown in Figure 3, the behavior problem in the dyslexic group was correlated with paternal rejection (r = −0.33, *p* < 0.05) and paternal anxious rearing (r = −0.33, *p* < 0.05). Moreover, a negative correlation was observed between the score for popularity and the score for paternal (r = −0.31, *p* < 0.05) and maternal rejection (r = −0.32, *p* < 0.05). In the relationship between physical appearance and parental rearing style, a moderate and positive correlation was found between physical appearance and maternal emotional warmth (r = 0.36, *p* < 0.05). No statistically significant correlations were found between other aspects of self-concept and parental rearing styles. The above results indicated that some domains of self-concept in children with dyslexia were related to parental rearing styles.

## 4. Discussion

To our knowledge, the present study was the first to investigate the self-concept, parental rearing styles and their relationship in Chinese dyslexic children. We investigated the demographics, self-concept and parental rearing styles of children in the dyslexia group and the control group and found some thought-provoking results. By using our self-developed auxiliary diagnosis tool, named CRAT, we found that the language processing ability of dyslexic children was obviously worse than that of normally developing children. In terms of self-concept, the academic competence self-concept, popularity self-concept and general self-concept of the dyslexic children were lower than those of the control group, in accordance with our hypothesis. The number of dyslexic children with low self-concept was more than that in the control group. For parental rearing style, compared with the control group, the results showed that fathers of children with dyslexia were less protective and less anxious in rearing style, and mothers of children with dyslexia were nor over-anxious in their rearing style. Correlation analysis also showed that some of the parental rearing styles were related to the self-concept of dyslexic children. In addition to the main results mentioned above, we also found that children with dyslexia had difficulty in doing homework and were scolded by their parents in significantly higher numbers than the control group. Moreover, there was also a statistical difference in the distribution of maternal educational level and monthly family income between the two groups. Based on multivariate linear regression, we found that the place of residence and participation in physical activity may be influential factors on self-concept in children with dyslexia.

Several studies have conducted investigations into the influencing factors facing dyslexic children in China. Consistent with our findings, Xue et al., found that there were statistical differences in monthly family income and maternal education level between the dyslexic and non-dyslexic children, which matched on gender, age and grade [32]. Similarly, maternal education level has been reported to be associated with dyslexia in a cross-sectional epidemiological study [33]. However, the study from Guangzhou could not find such results, but observed a statistical difference in paternal educational level between dyslexic and non-dyslexic [6]. As other studies have widely suggested, education and income were determinants of socioeconomic status, and socioeconomic status is linked to children’s language development [34]. Lower parental educational levels, as well as poor family income, may contribute to a less favorable environment for learning, affecting the occurrence of dyslexia. In addition, mothers in China were often the primary caregivers of children. The communication between mothers and children was more frequent than that between children and fathers. Hence, the influence of mothers’ educational level on children may be greater than that of fathers.

It is very important to explore the risk factors and prevent the occurrence of dyslexia, but for these children already suffering from dyslexia, the importance of promoting their self-concept cannot be ignored. Nowadays, a growing body of research is paying attention to the self-concept of children, whereas the research on self-concept in dyslexic children is absent [13,35,36]. Many studies have shown that children with attention deficit hyperactivity disorder (ADHD) have a lower self-concept than normal children [36]. In addition to ADHD, anxiety was often reported in children with learning difficulties. These disorders have been shown to affect children’s self-concept and lead to adverse emotional and social problems [37]. In view of the high comorbidities of dyslexia and other disorders affecting children’s self-concept, we investigated the self-concept of children with dyslexia only, in order to exclude the influence of other comorbidities. Just as in children with ADHD, the self-concept of children with dyslexia was easy to become lower due to the negative feedback from themselves and the external environment in certain dimensions. McArthur et al., have shown that academic self-concept and general self-concept were reduced in poor readers, which is consistent with our findings [17]. Teachers and peers often have a negative view of the academic and social abilities of children with learning problems [38], which may also contribute to a decline in the popularity self-concept of children with dyslexia. Nevertheless, inconsistent results have also been reported, with one study indicating a higher self-concept in dyslexic children than in non-dyslexic children [39]. Such results may be due to the mismatch between the case group and the control group. Age and gender are known to affect the self-concept. There is evidence that self-concept fluctuates throughout the life cycle, declining gradually from childhood through adolescence, and then rising again in adulthood [40]. Katzir et al., indicated that gender may influence self-concept, according to the low academic self-concept in females found in their study [41]. However, our study had already matched the corresponding control group based on age, gender and grade in the design stage, and well as eliminating these two confounding factors. Therefore, our results are relatively reliable. In the multivariate linear regression analysis of the self-concept of children in dyslexia, we found that children living in rural areas have higher self-concept than those living in urban areas. We hold the opinion that the higher self-concept of rural dyslexic children was probably due to the low academic requirements of parents and teachers. Nowadays, students in urban area are in fierce competition in their studies. The high pressure of examinations and competition, and the high expectation of parents and teachers, will both affect the self-concept of children with dyslexia. The point is that children with dyslexia are unable to learn normally as well as other children. Therefore, they not only have to suffer from the pressure of their study, but also the misunderstanding of their parents, teachers and peers. All of these phenomena lead to a decline in their self-concept. Compared with urban areas, the learning environment in the rural areas of Shantou is more relaxed. The parents and teachers have lower requirements on children’s academic performance. In addition, academic failure in rural children with dyslexia is often considered a normal phenomenon because of stereotypes in rural areas. Consequently, the place of residence may be an influential factor in the self-concept of children with dyslexia. The self-concept of rural dyslexic children may be higher than that of urban dyslexic children. Another influence factor found in our study was participation in physical activity. Our results showed that this may enhance self-concept in dyslexic children. According to a study conducted by Vella et al., positive physical activity could improve general self-concept, which may also support the psychosocial development of children [42]. The exercise and self-esteem model also showed that taking part in the physical activity could increase physical self-concept, which ultimately increases general self-concept [43].

In addition to these influential factors on self-concept in children, external factors like parental rearing style and peer relationship can also affect self-concept. Among these, the parental rearing style was regarded to have a great influence on the development of children’s self-concept [44]. Based on the importance of parental rearing style, we investigated the parenting style of dyslexic children in Shantou. We found an interesting result that, compared to normal parents, the fathers of dyslexic children were less overprotective and anxious in their rearing style. Similarly, mothers of dyslexic children were also less anxious than normal parents in their rearing style. These results may represent an indifferent and neglectful attitude of parents toward children with dyslexia. According to Maccoby and Martin, parental rearing style can be divided into four types: authoritarian, authoritative or flexible, permissive and neglectful [45]. Studies have shown that parents who adopt neglectful rearing styles exhibited low requirements for their children, accompanied by a low level of responsiveness [46]. We suggest that the attitude of apathy and neglect towards children with dyslexia may be due to the long-term academic failure faced by children with dyslexia. The inability of children with dyslexia to improve their academic records has made most parents lose confidence and appear less protective and anxious than normal parents. In turn, this neglectful rearing style from parents can affect the cognitive and social abilities, academic achievement and mental health of children with dyslexia, resulting in a lower self-concept [47]. This may also partly explain our results, which indicated that paternal rejection, paternal anxious rearing and maternal rejection were negatively correlated with some dimensions of self-concept in the correlation analysis. We also found a positive correlation between maternal emotional warmth and the physical appearance self-concept of dyslexic children. This represented that more warmth and understanding children feel from their parents may help improve their self-concept [48,49]. To our knowledge, most research on self-concept now focuses on adolescence [50]. No research has been conducted on the relationship between the self-concept of dyslexic children and their parental rearing styles. Due to the particularity of dyslexia, it is very important to pay attention to the self-concept of dyslexic children. We advocate regular mental health assessment and timely psychological intervention for dyslexic children. Parents of children with dyslexia may also need some psychological intervention and parenting guidance, which is very important for the growth of their children.

Our study had some limitations. Although we mitigated the effects of confounders as much as possible through the matching method, there are still some influence factors affecting the self-concept in children. The results of parental rearing style were objectively evaluated by children, and there may be some recall bias. Finally, the dyslexic children in the current study were all from Shantou Mental Health Center, with only a general representativeness. Therefore, the interpretation of the current results should be cautious. Future studies should explore further possible influencing factors which can provide clues for improving the self-concept of children with dyslexia.

## 5. Conclusions

In general, the self-concept of children with dyslexia in Shantou was at a low level, and the parental rearing style played an important role in the self-concept of these children. Dyslexic children from rural areas demonstrated relatively higher self-concept than urban dyslexic children. The results from our study can provide reference for psychological intervention strategies for dyslexic children. It is beneficial for parents to adopt a proper rearing style to improve the self-concept of children with dyslexia, which can reduce the generation of negative emotions and behaviors in children.

## Figures and Tables

**Table 1:** General demographic characteristics of the study participants.

Variables	Dyslexic (*n* = 50)	Control (*n* = 50)	*χ*^2^/*t*	*p* ^‡^
Age, $\bar{x}\pm s$ ^†^	9.18 ± 1.37	9.18 ± 1.37	1.000	1.000
Gender, *n* (%)			1.000	1.000
Male	37 (74.0)	37 (74.0)		
Female	13 (26.0)	13 (26.0)		
Grade, *n* (%)			1.000	1.000
Grade 2	16 (32.0)	16 (32.0)		
Grade 3	15 (30.0)	15 (30.0)		
Grade 4	12 (24.0)	12 (24.0)		
Grade 5	7 (14.0)	7 (14.0)		
Place of residence, *n* (%)			3.273	0.070
Rural	10 (20.0)	3 (6.0)		
City	40 (80.0)	47 (94.0)		
Father’s educational level, *n* (%)			0.733	0.865
Junior high school or below	17 (34.0)	14 (28.0)		
High school or equivalent	13 (26.0)	15 (30.0)		
Bachelor’s degree or above	20 (40.0)	21 (42.0)		
Mother’s educational level, *n* (%)			8.695	0.034
Junior high school or below	15 (30.0)	16 (32.0)		
High school or equivalent	18 (36.0)	12 (24.0)		
Bachelor’s degree or above	17 (34.0)	22 (44.0)		
Monthly family income, *n* (%)			14.364	0.002
<5000	10(20.0)	8 (16.0)		
5000–10,000	27(54.0)	20 (40.0)		
>10,000	13(26.0)	22 (44.0)		
Single parent family, *n* (%)			0.571	0.450
Yes	5(10.0)	2(4.0)		
No	45(90.0)	48(96.0)		
Be scolded by parents, *n* (%)			43.022	<0.001
Yes	45 (90.0)	36 (72.0)		
No	5 (10.0)	14 (28.0)		
Family history of dyslexia, *n* (%)			2.250	0.134
Yes	4 (8.0)	0 (0.0)		
No	46 (92.0)	50 (100.0)		
Have difficulty in doing homework, *n* (%)			12.071	<0.001
Yes	47 (94.0)	33 (66.0)		
No	3 (6.0)	17 (34.0)		
Participate in physical activity, *n* (%)			0.042	0.838
Yes	31 (62.0)	33 (66.0)		
No	19 (38.0)	17 (34.0)		

^†^: The mean ± standard deviation is expressed as $\bar{x}\pm s$. ^‡^: *p* values were calculated from paired *t* test for continuous variable, McNemar test for the binary variable and Bowker test for multi-categorical variable.

**Table 2:** Comparison of CRAT between the dyslexic group and the control group.

Variables	Dyslexic (*n* = 50)	Control (*n* = 50)	*t*	*p* ^#^
Phonological awareness				
Tone scores ^†^	8.32 ± 3.24	10.04 ± 3.12	2.876	0.006
Onset scores	7.96 ± 2.28	9.26 ± 1.81	3.168	0.003
Rime scores	7.00 ± 2.80	8.48 ± 2.40	2.913	0.005
Total scores	23.28 ± 6.62	27.78 ± 5.79	3.625	<0.001
Morphological awareness				
Chinese word formation time (s) ^‡^	182.4 ± 42.66	146.6 ± 31.02	4.866	<0.001
Chinese word formation scores	9.22 ± 1.27	9.86 ± 0.64	8.490	<0.001
Rapid automatized naming				
Time (s) ^‡^	19.15 ± 4.34	13.68 ± 2.91	9.513	<0.001
Number of wrong words in reading	0.53 ± 1.06	0.15 ± 0.38	2.293	0.026
Orthographic awareness				
Non-character recognition scores	15.14 ± 2.04	16.54 ± 1.43	3.612	<0.001
Radical position time (s) ^‡^	38.50 ± 12.87	30.47 ± 11.49	3.326	<0.001
Radical position score	10.12 ± 1.71	11.26 ± 1.31	3.384	<0.001
Reading ability				
Number of words in 1 min of reading	170.40 ± 39.56	219.40 ± 44.48	5.500	<0.001
Time for reading an article (s) ^‡^	116.60 ± 51.85	87.01 ± 36.13	3.220	0.002
Total score of reading comprehension	10.01 ± 2.24	11.06 ± 1.87	2.493	0.016

^†^: The scores were expressed as mean ± standard deviation ($\bar{x}\pm s$). ^‡^: Time was measured in seconds (s). ^#^: *p* values were calculated from paired *t* test for continuous variable.

**Table 3:** Comparison of different self-concept levels between dyslexic group and control group.

Levels, *n* (%)	Dyslexic (*n* = 50)	Control (*n* = 50)	*χ* ^2^	*p* ^†^
			15.348	0.002
Low ^‡^	13 (26.0)	5 (10.0)		
Normal	18 (36.0)	19 (38.0)		
High	19 (38.0)	26 (52.0)		

^†^: *p* values were calculated from Bowker test (an extension of the McNemar test). ^‡^: There was a statistical difference in the composition ratio of children with low level of self-concept between two groups (*p* < 0.05).

**Table 4:** Multivariate linear regression analysis of influencing factors on self-concept in dyslexic group (*n* = 50).

Variables ^†^	*β*	*SE*	*t*	*p*
Participate in physical activity ^†^	0.36	2.72	2.81	<0.01
Place of residence ^†^	−0.32	3.20	−2.46	<0.05
Paternal educational level	−0.02	3.62	−0.11	0.92
Maternal educational level	0.10	4.21	0.46	0.65
Monthly family income	−0.07	3.51	−0.43	0.67
Have difficulty in doing homework	−0.09	6.71	−0.27	0.79

Abbreviations: *SE*, standard error; *β*, standardized coefficients Beta. ^†^: The multiple linear stepwise regression models only included participation in physical activity and place of residence, and excluded age, gender, single parent family, parental educational level, being scolded by parents and having difficulty in doing homework. The adjusted *R*^2^ = 0.27, *F* = 9.93, *p* < 0.001.

## Data Availability

The data presented in this study are available on request from the corresponding author. The data are not publicly available due to the requirements of the written informed consent.
